# Chloral in Delirium Tremens

**Published:** 1872-03-01

**Authors:** Chas. W. Earle

**Affiliations:** Physician to Washingtonian Home, Chicago; 673 and 675 West Lake


### CHLORAL IN DELIRIUM TREMENS.

BY CHAS. W. EARLE, M. D., PHYSICIAN TO WASHINGTONIAN HOME, CHICAGO.

Chloral, although discovered thirty years ago by Liebig, was not introduced to the notice of the profession until 1869. It is the result of chlorine on alcohol—anhydrous (without water) it is an oily, pungent fluid. Combined with water, which it does readily, it becomes the chloral hydrate.* Since the introduction of this drug to the notice of the medical profession in this country, it has been used in nearly every disease, in large and small doses. We probably do not know at this time its exact worth as a remedial agent, but clinical experience is helping us form a correct estimate. Having used it for more than fifty cases of delirium tremens, and the excitement produced by alcoholic stimulants, I wish briefly to give the results. At least two indications present themselves for treatment in the majority of patients admitted to our Home, viz.—to give nourishment and procure sleep. The first is best met by giving beef tea, milk and eggs, etc., etc.—in a word, concentrated nutriments which are easily digested. The stomach is not in a condition to digest solid food, and we therefore give the liquid nourishment. To quiet the extreme nervousness during the day, previous to giving chloral, we are in the habit of giving bromide of calcium, bromide of potassium, valerian, etc. A combination of the bromides of calcium and potassium with cannabis indica is excellent. But surpassing them all, when night comes and we wish to give our patient sleep, is the chloral. It matters little how it is given. It is not as necessary in the cases we treat at the Home to disguise the taste of the drug as in private practice. The sharp, pungent taste, to one habituated to the use of *ordinary whisky*, is not particularly unpleasant. Ffteen grain doses repeated often, until the required result is obtained, seem to give a better effect than to give larger doses.

Case 6. T. T.—aet. 33. Admitted Sept. 21, with delirium tremens. Placed immediatlely on chloral; gave 100 grs. from 7 o'clock in the evening until midnight; at this time he fell asleep and waked at 8 in the morning perfectly rational. During the day he made his escape from the Home, and came in during the evening drunk, but not with the delirium. Slept after the administration of 40 grs. of chloral; convalesced on tonics, and is now doing well; boards at the Home, and is working at his trade.

The exhibition of morphine with chloral seems, in some cases, to give a better result than either drug given singly. The following formula, of which a teaspoonful may be given every two hours, has given decided benefit:

| | | |
|---|---|---|
| ℞ | Chloral Hydrate | ʒ ii. |
| | Morphiæ Acet. | grs. ii. |
| | Syrupi Zinziberis | ℥ ii. |

M.

Patients on the verge of delirium taking this for ten or twelve hours, and followed with somewhat larger doses of chloral, very frequently escape the threatened attack, and make a rapid recovery.

Case 8. J. E.—aet. 31. Had some family difficulty and took to drinking. He came very near having delirium, and consulted Dr. Davis. Was admitted to the Home on substantially the prescription given above. The chloral was given at night, which procured a moderate night's rest. A continuation of the same treatment for two or three days brought him to his former condition, and he was discharged to engage in business.

Case 45—in private practice—K.—aet 27. Has drank moderately for several years; never has been in the habit of getting intoxicated; within the last year, however, his drinking has affected him very strangely—it makes him crazy; after a few drinks he is unable to remember anything that transpires—everything is a blank for thirty or forty hours. A few days since he left his residence at 1 p. m., and drank mixed liquors during the afternoon. He returned in the evening perfectly crazy, wild and boisterous. His wife gave him 15 grs. chloral and $\frac{1}{8}$ gr. morphia, up to the time I saw him, at 8 p. m. I then gave him 10 grs. chloral; at 9 o'clock, 25 grs.; at 10 o'clock, 25 grs. He was now quiet and slept most of the time. At 11 o'clock he waked enough to take 10 grs. more; at 12 o'clock he was very quiet, and I left him in charge of his wife. He seemed to realize, however, that I had gone, and began to be more boisterous. His wife, up to 2 o'clock in the morning, gave him 75 grs. more of the chloral, when he sank into a quiet sleep and rested till 7 a. m., when he roused sufficiently to take a small amount of fluid nourishment, and then slept until noon. The next night he took a small quantity of

*Wood & Bache, 1870.

chloral, and rested quietly. He was now ordered a mild bitter tonic, and made a rapid recovery.

No accident has occurred, nor any unfavorable result followed the use of chloral. One patient took 200 grs. from 11 o'clock p. m. to 6 a. m. The sleep which followed, for about 10 hours, was in the main very natural. He breathed stertorously, however, for a short time.

An inmate of the Home (an opium eater) feeling badly one morning procured, without the knowledge of the Superintendent, and took at one dose, what I compute to be at least 150 grains. He was unconscious an hour; breathed with difficulty; pupils contracted. He was roused occasionally when his breathing became very labored, and finally came to himself just as I arrived to make my daily visit.

673 and 675 West Lake.

